# Semaglutide and Tirzepatide reduce alcohol consumption in individuals with obesity

**DOI:** 10.1038/s41598-023-48267-2

**Published:** 2023-11-28

**Authors:** Fatima Quddos, Zachary Hubshman, Allison Tegge, Daniel Sane, Erin Marti, Anita S. Kablinger, Kirstin M. Gatchalian, Amber L. Kelly, Alexandra G. DiFeliceantonio, Warren K. Bickel

**Affiliations:** 1 Fralin Biomedical Research Institute at VTC, Virginia Tech, Roanoke, VA USA; 2 Graduate Program in Translational Biology, Medicine, and Health, Blacksburg, VA USA; 3 Virginia Polytechnic Institute and State University, Blacksburg, VA USA; 4 Virginia Tech Carilion School of Medicine, Roanoke, VA USA

**Keywords:** Human behaviour, Translational research

## Abstract

Alcohol Use Disorder (AUD) contributes significantly to global mortality. GLP-1 (Glucagon-like peptide-1) and GLP-1/GIP (Glucose-dependent Insulinotropic Polypeptide) agonists, FDA-approved for managing type 2 diabetes and obesity, where the former has shown to effectively reduce the consumption of alcohol in animal models but no reports exist on the latter. In this report, we conducted two studies. In the first study, we conducted an analysis of abundant social media texts. Specifically, a machine-learning based attribution mapping of ~ 68,250 posts related to GLP-1 or GLP-1/GIP agonists on the Reddit platform. Secondly, we recruited participants (n = 153; current alcohol drinkers; BMI ≥ 30) who self-reported either taking Semaglutide (GLP-1 agonist), Tirzepatide (the GLP-1/GIP combination) for ≥ 30 days or, as a control group; no medication to manage diabetes or weight loss for a within and between subject remote study. In the social media study, we report 8 major themes including effects of medications (30%); diabetes (21%); and Weight loss and obesity (19%). Among the alcohol-related posts (n = 1580), 71% were identified as craving reduction, decreased desire to drink, and other negative effects. In the remote study, we observe a significantly lower self-reported intake of alcohol, drinks per drinking episode, binge drinking odds, Alcohol Use Disorders Identification Test (AUDIT) scores, and stimulating, and sedative effects in the Semaglutide or Tirzepatide group when compared to prior to starting medication timepoint (within-subjects) and the control group (between-subjects). In summary, we provide initial real-world evidence of reduced alcohol consumption in people with obesity taking Semaglutide or Tirzepatide medications, suggesting potential efficacy for treatment in AUD comorbid with obesity.

## Introduction

Alcohol Use Disorder (AUD) is a multi-faceted chronic relapsing brain disorder and contributes greatly to global mortality, yet it remains one of the most undertreated conditions. The only FDA-approved treatments (disulfiram, naltrexone, and acamprosate) have been modestly successful in treating AUD (e.g., naltrexone reduces relapse rates by 5%) (for a detailed review and meta-analysis^1,2^), but still are severely under-prescribed. Further, 2/3 of patients relapse within the first year in abstinence-based treatment models^3^. A meta-analysis reported that, at maximum, 50% of individuals diagnosed with AUD achieve remission during extended follow-up periods^4^. Therefore, a crucial need for novel and effective AUD treatments still remains.

Incretin hormones (such as Glucagon-like peptide; GLP-1 and glucose-dependent insulinotropic polypeptide; GIP), produced in the intestine and brain in response to nutrients in the gut, are responsible for maintaining glycemic control^5^. Shared neural mechanisms between the food reward system and AUD pathways are well established^6–9^. The ventral tegmental area (VTA) and nucleus accumbens (NAc), key brain areas involved in the reinforcing effects of food and alcohol abuse, contain GLP-1 receptors, indicating central mediation of GLP-1 agonists^10^.

GLP-1 peptides, FDA-approved for type-2 diabetes (e.g., Semaglutide and Exenatide) and weight loss (Semaglutide) have been studied for their effects on alcohol use in both preclinical and clinical studies (for a detailed review: 10). GLP-1/GIP peptide (Tirzepatide) has also been FDA-approved for treating type-2 diabetes. However, to our knowledge, there have been no research studies to investigate the effects of Tirzepatide on alcohol consumption. To date, only one clinical trial has investigated the effects of a GLP-1 agonist, exenatide, on individuals seeking treatment for AUD^11^. Although exenatide did not significantly reduce days of heavy drinking in the experimental group, fMRI revealed a significant attenuation of activity in the ventral striatum and septal area when images of alcohol were shown in the experimental group. Further analysis revealed a substantial reduction in heavy drinking in patients with obesity in the experimental group. To note, placebo effects in the group with BMI < 25 also showed a significant reduction in heavy drinking days. Additionally, there is strong preclinical evidence that GLP-1 agonists reduce alcohol intake in rats, mice, and vervet monkeys^8,10,12–14^, The administration of the GLP-1 agonist Exendin-4 (Ex-4) suppressed accumbal dopamine release in response to alcohol^8^ and microinjection of Ex-4 to the nucleus accumbens or ventral tegmental area decreases alcohol intake, suggesting a possible central mechanism^15–17^. However, the administration of exendin-9–39, a GLP-1R antagonist, resulted in elevated alcohol consumption in rats^7^. Reduced alcohol intake, relapse-like drinking and attenuated alcohol-induced locomotor stimulation in mice and rats have been reported by administering semaglutide, one of the most commonly prescribed GLP-1 agonists^12,18,19^. Furthermore, other GLP-1 agonists, such as liraglutide^20^, dulaglutide^21^, and AC3174^22^ have been shown to reduce alcohol-related behaviors. Although there is no literature on Tirzepatide, the strong mechanistic link between GIP and GLP-1 physiologically^23^ warrants an investigation on alcohol use. Additionally, there is no evidence about the difference of GLP-1 effects on the severity of alcohol use. However, given the cumulative evidence in humans and animal models, the influence of GLP-1 agonists on alcohol-related behaviors cannot be denied.

While administration of GLP-1 agonists has convincingly shown to reduce alcohol intake in preclinical models, more clinical data is crucial to understanding the efficacy and mechanism of several GLP-1 agonists and other related drug classes (e.g., Tirzepatide) on alcohol intake. In this article, we systematically analyzed social media discussions centered around GLP-1 agonists to generate insights and characterize the real-world evidence regarding effects on alcohol consumption in a substantial number of social media texts, replicating the workflow from^24^ (Suppl. Fig. 1). Next, to gain detailed information on the impact of GLP-1 medication on alcohol consumption, we surveyed individuals with and without Semaglutide or Tirzepatide medications and assessed their historical and contemporary self-reports of alcohol usage and its direct effects. First, for the social media analysis, we retrieved all posts containing keywords related to GLP-1 agonists from Reddit, and categorized them by employing a machine learning algorithm to identify major themes of discussions. Next, we identified all alcohol-related posts and categorized them by impact on alcohol. Second, for the detailed reports on alcohol usage, we conducted a between and within participant remote survey among individuals taking Semaglutide or Tirzepatide or neither of these medications, who had a BMI greater than 30 and drank alcohol. Note heretofore, there have been no other reports of Tirzepatide effects on alcohol consumption. We hypothesize reduced alcohol intake and less impact of the self-reported effects of alcohol, in individuals on Semaglutide or Tirzepatide medications, as compared to those not taking these medications.

## Results

### Study 1: social media analysis

#### Optimum clusters identification

**Figure 1:**
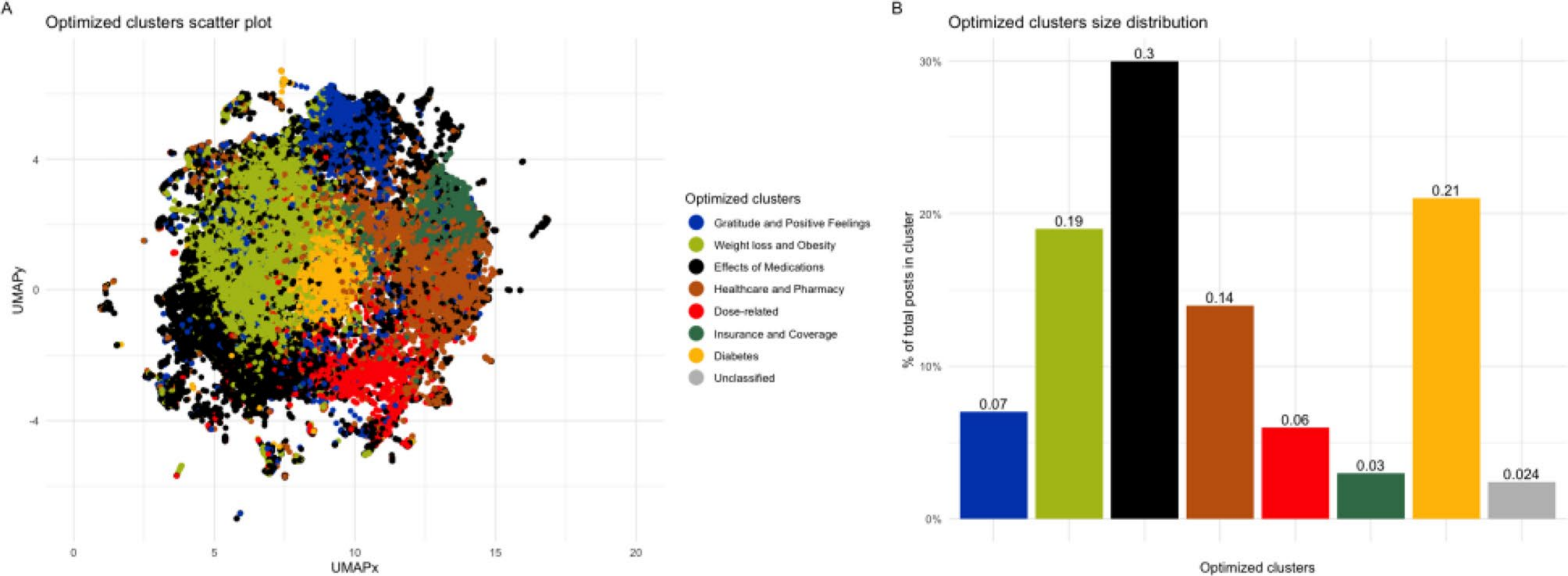
Final optimized clusters. Shown are the final optimized clusters obtained for our sample. (**A**) a scatterplot visualized by the UMAP model, we see the two UMAP components denoted as UMAPx and UMAPy represent the x and y axis of the scatter plot, with different colors to show the different clusters. (**B**) the distribution of posts into each of these clusters with corresponding colors.

Using the two-step approach for cluster identification, 13 optimum clusters were identified by centroidal *k*-means clustering; as the trendline of the distortion score shows a steep fall before becoming linear-like at 13 (Suppl. Fig. 2). Figure 1A shows the 2-D visualization of the clusters using UMAP. Further inspection of the unigrams and bigrams of the top 50 keywords in each cluster revealed similar underlying themes, such as availability of medications in pharmacies and doctors’ prescriptions were merged together in “Healthcare and Pharmacy”. Therefore, we created 8 final clusters by merging similar clusters. Overall, 8 notable themes emerged from the discussions of GLP-1 related posts on reddit (Fig. 1B). Of these, discussions related to “Effects of Medications” (30%), “Diabetes” (21%), “Weight loss and Obesity” (19%) and “Healthcare and Pharmacy” (14%) dominated (Fig. 1B).

#### Important underlying features of optimized clusters

**Figure 2:**
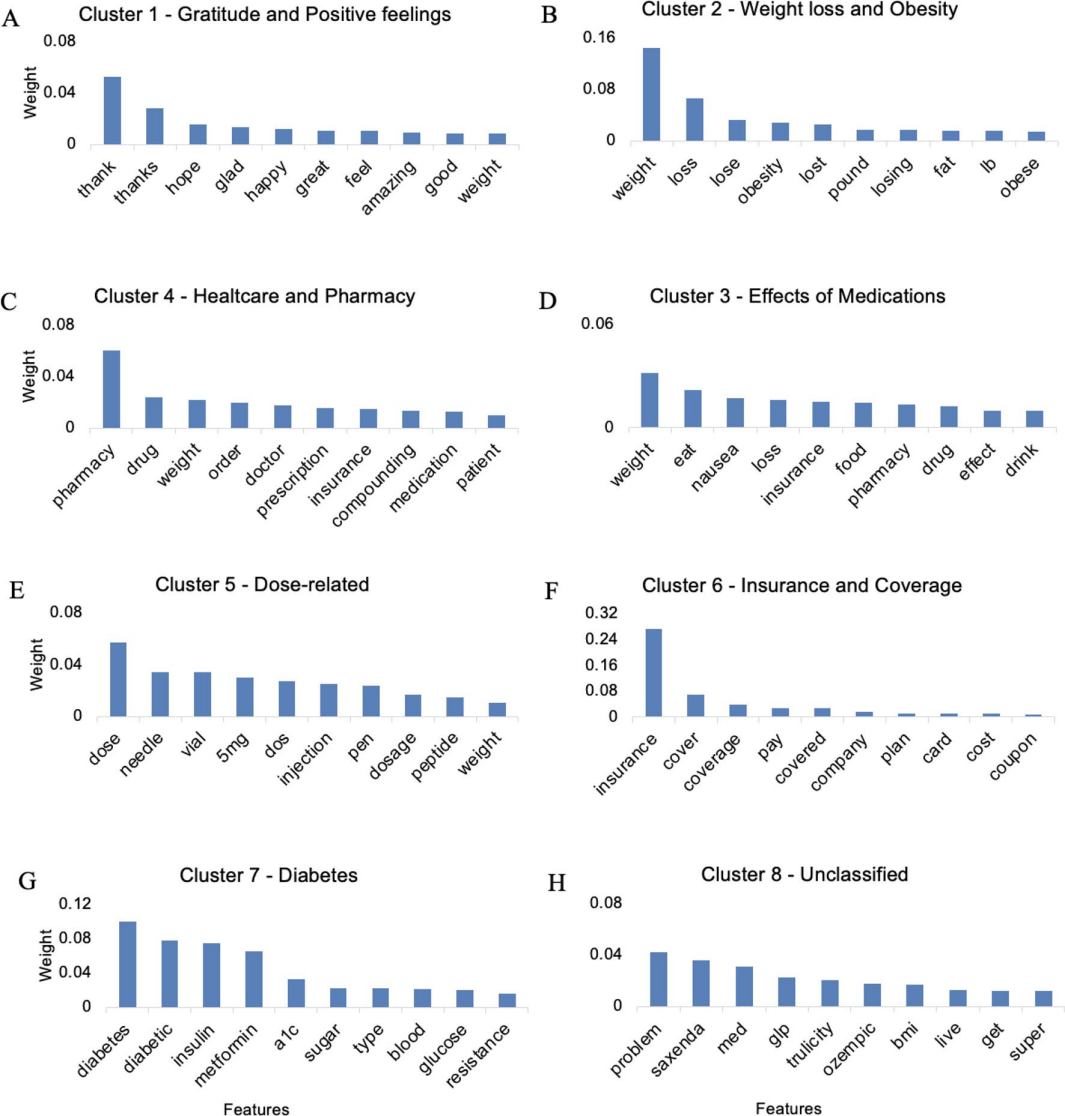
Underlying features obtained by training a supervised learning-based Random forest binary classifier. All clusters are identified by unique underlying features, as evident by the output (**A**–**H**).

A random forest based binary classifier was trained to obtain the underlying features and their respective weights. The results indicate unique underlying features for each theme; a) “thanks”, “hope” and “glad” are the most weighted features in Gratitude and Positive feelings, (Fig. 2A,B) “weight”, “lose” and “obesity” in Weight loss and Obesity, (Fig. 2B,C) “weight”, “eat”, “nausea” and “drink” are most notable in the effects of medications, (Fig. 2C,D) “pharmacy”, “drug”, and “doctor” in Healthcare and Pharmacy, (Fig. 2D,E) “dose”, “needle”, “vial” and “5 mg” in Dose-related discussions (Fig. 2E,F) “insurance”, “cover” in Insurance and Coverage, (Fig. 2F,G) “diabetes”, “diabetic”, “insulin” in Diabetes (Fig. 2G,H) “problem”, “med” in the Unclassified cluster (Fig. 2H). Overall, “weight”, “diabetes”, “pharmacy”, “dose” and “insurance” dominate the discussion among all identified themes.

#### Data representation and exploration

To further explore the discussions in the themes identified through the binary classifier, we visualized the top 30 unigrams; single words (Suppl. Fig. 3) and bigrams; two words (Suppl. Fig. 4) among our samples. The keywords in unigrams as well as bigrams disclosed that the identified themes broadly captured the related discussions; albeit with some overlap. For example, keywords such as weight, loss and weight loss were noted in all distinct clusters. Interestingly, effects of medications had discussions pertinent to suppression of appetite and reduced food cravings; with “don’t think”, “don’t want” and “food noise” observed as frequent keywords.

**Figure 3:**
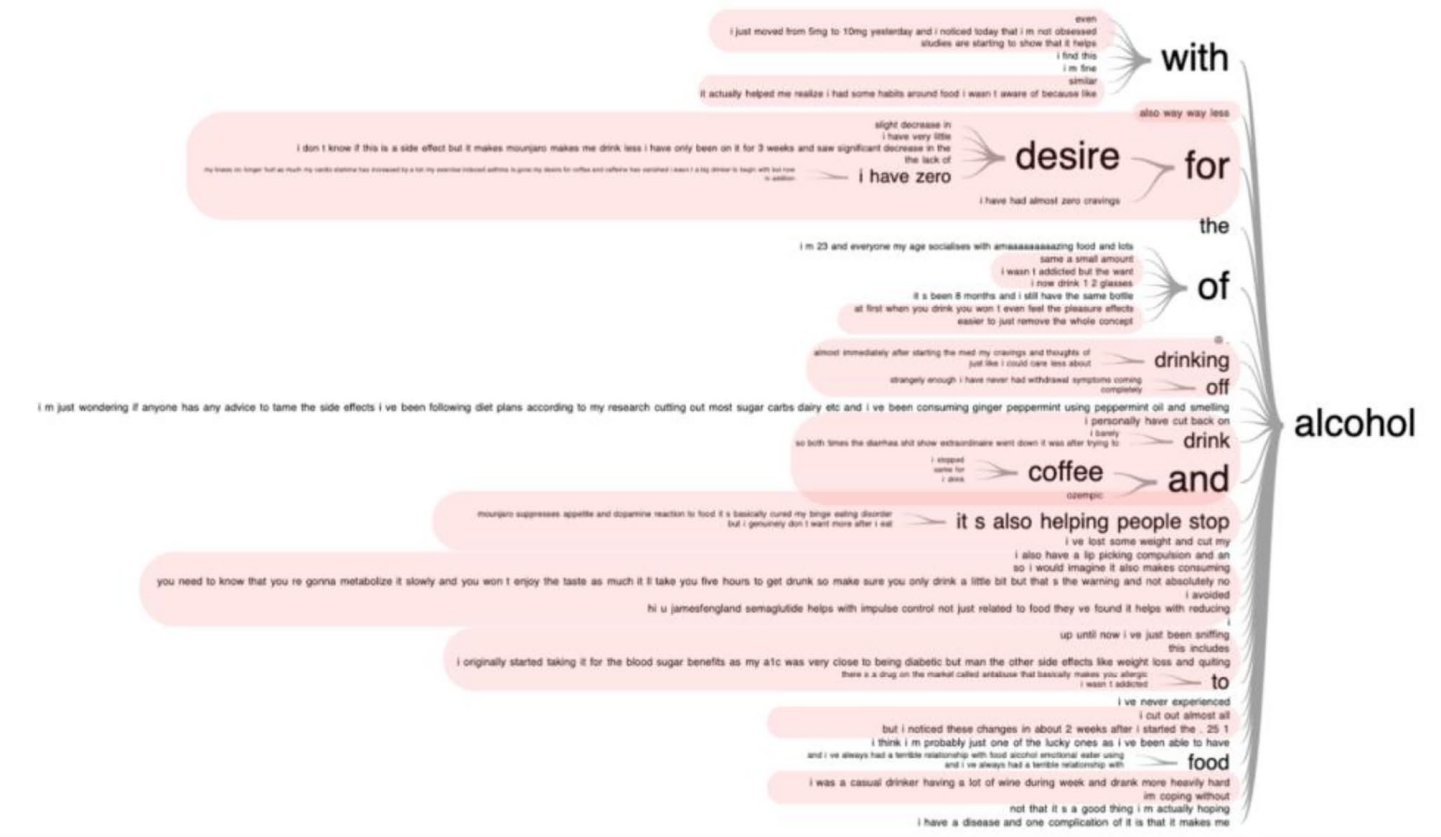
Alcohol related word tree. Prefixes from effects of medications cluster generated for the keyword “alcohol”. Any phrases related to a decrease/change in alcohol consumption or effects on alcohol are highlighted with red. It is evident that most alcohol-related posts point towards reduced alcohol usage.

**Figure 4:**
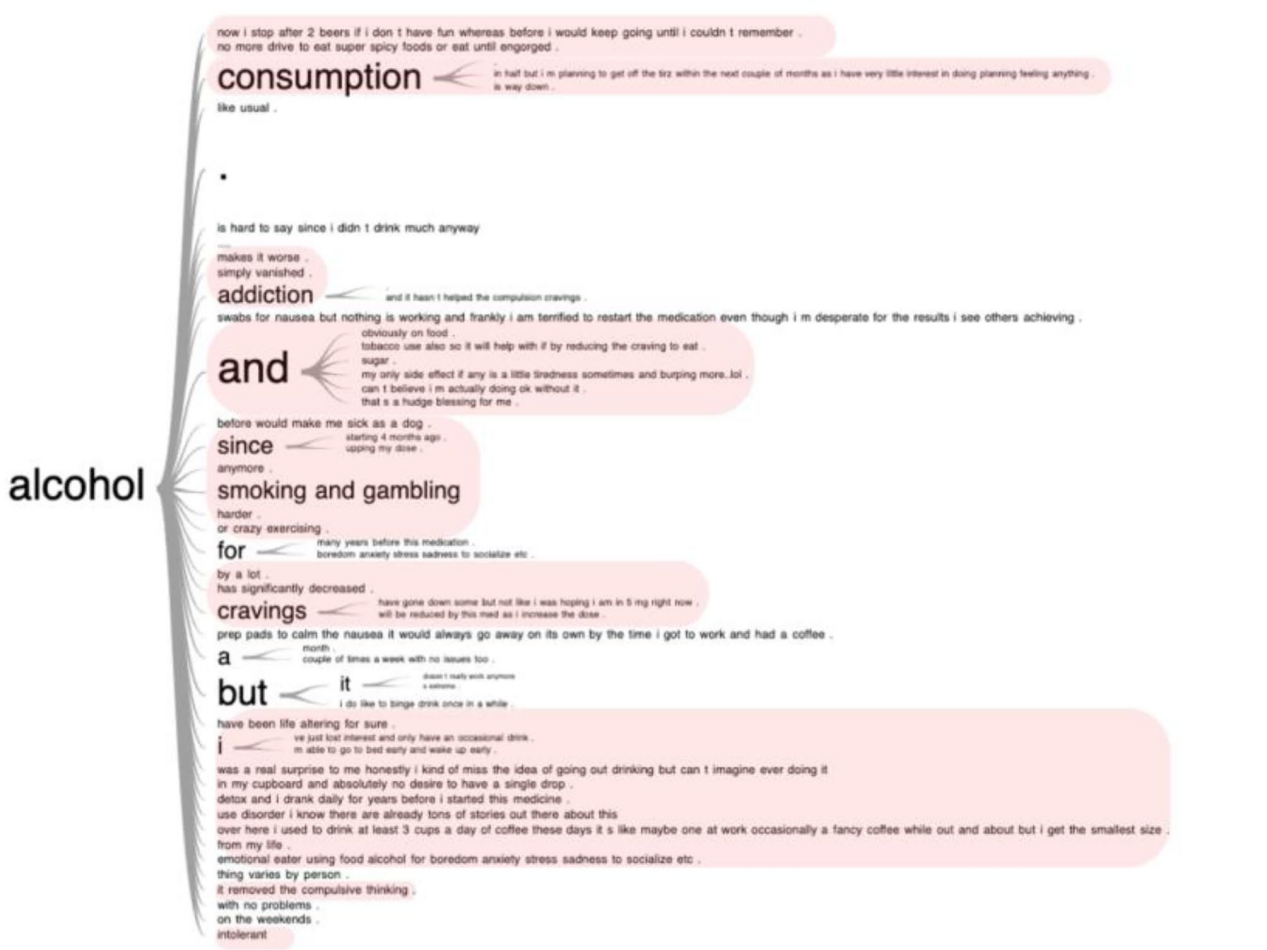
Alcohol related word tree. Suffixes from effects of medications cluster generated for the keyword “alcohol”. Any phrases related to a decrease/change in alcohol consumption or effects on alcohol are highlighted with red. It is evident that most alcohol-related posts point towards reduced alcohol usage.

Case studies and news articles have pointed towards an unexpected side effect of GLP-1 medications, i.e. suppression of addictive behaviors such as reduction of food noise and loss of desire to consume alcohol^25–28^. To further investigate the effects of GLP-1 medications on alcohol consumption, we made word trees to visualize the discussions related to alcohol within each cluster. Alcohol-related discussions did not emerge as a distinct theme in our main analysis, but on inspection were found to be spread throughout the clusters. We performed a functional enrichment analysis to investigate if specific clusters overly represented alcohol posts. Most of the alcohol-related posts were over-enriched in two clusters: cluster 3; effects of medications (n = 826, p < 0.001), and cluster 2; weight loss and obesity (n = 439, p = 0.002). Usage of alcohol is represented by word trees in Cluster 2 and 3 (Figs. 3, 4, Suppl. Fig. 5); highlighted portions of text indicate posts related to the adverse effects or reduced usage of alcohol, e.g. I have zero desire to drink, alcohol consumption is way down, cravings are gone etc. 962 individuals made a total of 1580 alcohol related posts. Most notably, 71.7% (1134/1580) of the alcohol posts addressed reduced cravings, reduced usage and other negative effects due to drinking, containing keywords such as stopped, reduced, sick, full, nausea, don’t want, cutback, low tolerance, craving. On manual inspection of randomly selected alcohol-related posts, we confirmed that these were unique individuals posting about their own or closely-known experiences.

#### Full order partial correlation analysis

**Figure 5:**
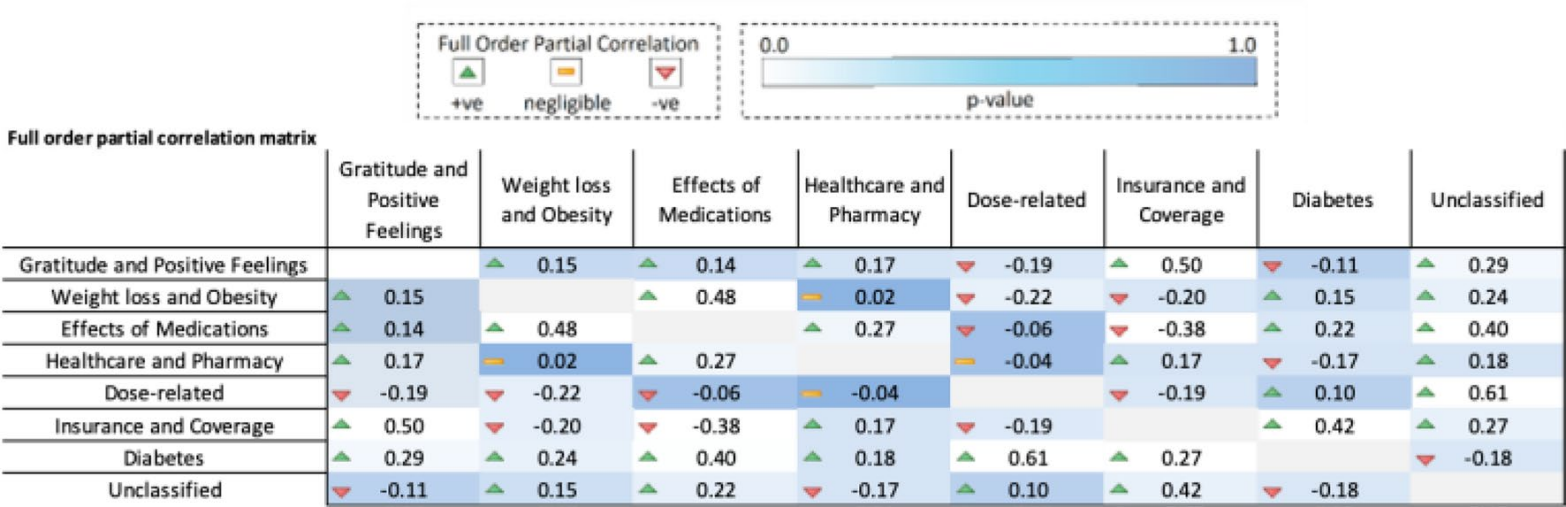
Optimum clusters full order partial correlation matrix with p values. Figure shows 8 unique GLP-1 post themes. Green arrows indicate a positive correlation between themes while a red arrow indicates a negative correlation between themes. The background color represents the p value, ranging from 0: white, to 1: dark blue.

First, we calculated the proportions of posts on each time period in each cluster, organizing the data into a time series output. Full order partial correlation analysis on the time series output provided an insight into the strength of the relationship between the 8 identified optimum clusters (Fig. 5). We observed a majority of positive correlations among our clusters, indicating an uptick in concurrent discussions relevant to all themes e.g., gratitude and positive feelings have a significant positive correlation with all clusters, e.g., Insurance and coverage (R = 0.50, p < 0.001) and Diabetes (R = 0.29, p > 0.05) except Dose-related (R = − 0.19, p > 0.05). Most notable negative correlation was observed between effects of medication and Dose-related (R = − 0.06, p > 0.05) and Insurance and coverage (R = − 0.38, p < 0.05) discussions, indicating that there is a shift from discussing effects of medications to seeking information regarding doses and acquiring the medication through insurance, as the medication gains popularity.

### Study 2: remote study

#### Cohort characteristics

We recruited 153 participants from various social media platforms to participate in our study. The demographics and cohort characteristics, none of which are significantly different between groups, are presented in Table 1. Briefly, a majority of participants were white, female, ~ 40 years old, with an average education of ~ 15 years and a mean BMI around 35. Individuals on Tirzepatide (n = 50) reported a median dose of 7.5 mg [range 0.5–15 mg], and on Semaglutide variants: Ozempic or Wegovy (n = 54) reported a median dose of 1 [range 0.25–2.4] and Rybelsus (n = 2) reported doses of 7 mg and 14 mg. Individuals and their medication group and doses are reported in Table 1 and Suppl. Table 1 respectively.

**Table 1:** Cohort characteristics of participants (N = 153).

	Control	Semaglutide	Tirzepatide	p-values
n	47	56	50	
Age [mean (SD)]	38.89 (11.29)	41.57 (9.08)	43.68 (8.22)	0.051
Education [mean (SD)]	15.39 (3.19)	15.00 (5.60)	15.40 (4.97)	0.887
Sex = male (%)	11 (23.4)	9 (16.1)	9 (18.0)	0.625
Ethnicity = not Hispanic or Latino (%)	42 (89.4)	53 (94.6)	48 (96.0)	0.377
Race (%)				0.395
Asian	0 (0.0)	0 (0.0)	1 (2.0)	
Black/African American	5 (10.6)	1 (1.8)	3 (6.0)	
Native Hawaiian or other Pacific islander	0 (0.0)	1 (1.8)	0 (0.0)	
Other	1 (2.1)	3 (5.5)	1 (2.0)	
White/Caucasian	41 (87.2)	50 (90.9)	45 (90.0)	
BMI [mean (SD)]	36.76 (6.69)	34.59 (5.69)	35.68 (6.80)	0.231
Medication (%)				–
Mounjaro (Tirzepatide)	–	–	50 (100.0)	
Ozempic or Wegovy (Semaglutide injection)	–	54 (96.4)	–	
Rybelsus (Semaglutide tablet)	–	2 (3.6)	–	
For weight loss (%)	–	51 (91.1)	36 (72.0)	–

#### Changes in alcohol consumption

**Figure 6:**
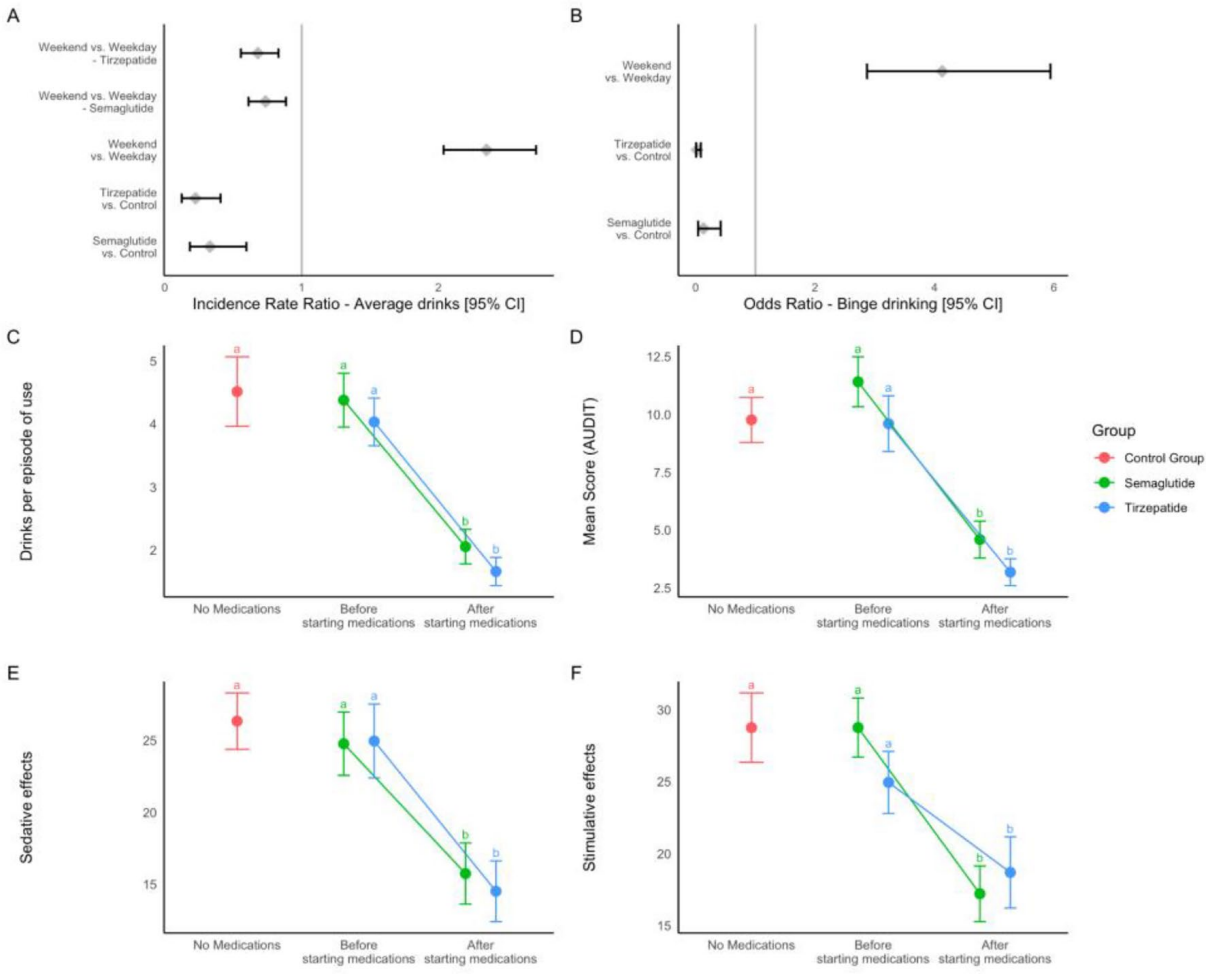
The remote study provides strong converging evidence of reduced alcohol use. (**A**) Incidence Rate Ratio of Average drinks calculated from the past 30 days Timeline Follow Back, as calculated by the lme4 package using the poisson distribution. (**B**) Odds Ratio of binge drinking over the past 30 days (binomial distribution, 0; no binge, 1; binge) Males (> 5 drinks), Females (> 4 drinks). (**A**, **B**) Grey diamonds represent the estimated incidence rate or odds ratio and the error bars are 95% confidence intervals. Findings are significant, since the 95% CI do not encompass 1. Comparison of (**C**) average drinks per episode of use (**D**) AUDIT scores (**E**) Sedative effects and (**F**) Stimulative effects of alcohol within and between groups. A significant reduction is seen both within (Before and after starting medications) and between (control vs. medication) groups for all four measures. Solid circles represent mean and the error bars are standard error of means. Significantly different points are denoted by different letters.

TLFB. We investigated between-subjects changes (control vs. medication groups) in alcohol consumption through the validated remote TLFB instrument. Participants drank significantly more on weekends than on weekdays (B = 0.85, SE = 0.07, p < 0.001). Individuals with obesity on Semaglutide (B = − 1.31, SE = 0.30, p < 0.001) or Tirzepatide (B = − 1.54, SE = 0.31, p < 0.001) had on average significantly fewer drinks than their counterparts not on any medication for diabetes or weight loss. Additionally, we found a significant two-way (Time x Group) interaction term in the model with lowest BIC, suggesting reduced drinking both on weekdays and weekends in the Semaglutide (B = − 0.17, SE = 0.10, p = 0.08) and Tirzepatide (B = − 0.45, SE = 0.10, p < 0.001), when compared to control (Fig. 6A). Secondly, we used the drinks per day of an individual to make a binomial distribution of binge drinking; individuals were classified as 1 and 0 for binge drinking (drinks = 5 + and drinks = 4 + for males and females respectively^29^). In the optimal binomial model, there was a significant main effect of Time i.e., weekend vs. weekday (B = 1.4178, SE = 0.1857, p < 0.001) and Group i.e., Semaglutide vs. Control (B = − 2.0517, SE = 0.6002, p < 0.001) and Tirzepatide vs. Control (B = − 3.7920, SE = 0.6764, p < 0.001; Fig. 6B), indicating that these medications reduce the odds of binge drinking. Overall, both average drinks and odds of binge drinking were found to be significantly lower in the medication groups i.e., Semaglutide and Tirzepatide, as compared to the control group.

AUDIT and drinks per episode of use. We report a main effect of Time (B = 6.63208, SE = 0.76773, p < 0.001), prior to and while on medications, indicating that AUDIT scores decreased after the participants started their current dose of Semaglutide or Tirzepatide (Fig. 6D). Complementary to change in TLFB and AUDIT scores, drinks per episode of regular use was also significantly lower after the participants started their current dose of their medication (B = 2.3443, SE = 0.2668, p < 0.001) (Fig. 6C). However, we did not find a dose-dependent change in average drinks or AUDIT scores in the medication groups, although a visual downward trend could be observed in the Tirzepatide group. (Suppl. Fig. 6). Finally, for the between group comparison, the current time point for both AUDIT (Semaglutide: B = − 5.0820 SE = 1.306, p = 0.001, Tirzepatide: B = − 6.6920, SE = 1.331, p < 0.001) and frequency per episode of use (Semaglutide: B = − 2.469, SE = 0.540, p < 0.001, Tirzepatide: B = − 2.838, SE = 0.552, p < 0.001) was significantly lower when compared to control group (Fig. 6C,D). In summary, we have strong converging evidence of reduced alcohol use in individuals taking Semaglutide or Tirzepatide.

#### The effects of alcohol intoxication are mitigated

We also investigated how the effects of alcohol differed in individuals with medication, prior to and while taking their Semaglutide or Tirzepatide medication. The repeated measures analysis revealed a significant main time effect i.e. prior to and while on the medications for both the stimulative (B = − 9.057, SE = 1.623, p < 0.001; Fig. 6E) and sedative effects (B = − 9.689, SE = 1.760, p < 0.001; Fig. 6F). Additionally, as a between-group analysis, we compared the control group to each medication group for both time points. The control group was significantly different than the current time point for both medications (Semaglutide: B = − 10.29, SE = 3.02, p = 0.004, Tirzepatide: B = − 11.445, SE = 3.08, p = 0.003), but was not different when compared to prior to starting each medication (n.s.). In summary, our results suggest that the effects of alcohol intoxication, particularly the stimulative and sedative effects, are reduced while taking these medications.

## Discussion

Here, we report a novel analysis of GLP-1 agonist and drinking related posts made to the social media platform reddit. Case studies and media articles have hinted at unexpected side effects of GLP-1 agonist medications, i.e. the suppression of addictive behaviors and desire to consume alcohol. The machine learning based analysis here provides preliminary empirical support of these phenomena by classifying and quantifying these experiences. We further report a reduction in alcohol consumption in a cohort of people self-reporting alcohol use and taking Semaglutide or Tirzepatide compared to controls. Participants endorsing Semaglutide and Tirzepatide use reported fewer drinks as measured by the TLFB, fewer binge episodes, and lower AUDIT scores compared to controls and compared to before starting their medication. Participants also reported less stimulating and less sedative effects of alcohol than before starting their medication and when compared to controls.

In contrast to self-reported surveys, passive observation has higher accuracy in areas where self-reporting doesn’t, such as recall area and social desirability bias^30^. By integrating both methodologies, we adopt a multi-faceted approach wherein the qualitative analysis corroborates the outcomes of our self-reported survey, thereby diminishing potential biases. In our case, we find that not only is there a self-reported decrease in alcohol use in individuals as evident by self-reports in our remote study but also a notable association of decrease in appetite of alcohol with individuals in the comprehensive qualitative social media analysis.

There are only 3 medications approved by the US Food and Drug Administration (FDA) to treat AUD: disulfiram, naltrexone, and acamprosate and they have poor compliance. Disulfiram acts by inducing nausea upon alcohol consumption, naltrexone is a mu-opioid receptor antagonist, therefore having broad action on the reward system, and the action of acamprosate is less well understood, but it is likely an allosteric modulator of GABAa receptors. Semaglutide and Tirzepatide are both GLP-1 receptor agonists, with Tirzepatide having additional action on and a higher affinity for glucose-dependent insulinotropic polypeptide (GIP) receptors.

The mechanism by which GLP-1 agonists reduce alcohol intake in humans is currently unknown, though a recent RCT reported reduced alcohol cue reactivity in the nucleus accumbens (NAc), a key region associated with alcohol’s rewarding properties, after treatment with the GLP-1 agonist Exenatide^11^. This is in accordance with preclinical studies demonstrating the ventral tegmental area and NAc as key areas mediating the effects of GLP-1 receptor agonists on alcohol intake^10,18^. Recent studies using fluorescently labeled semaglutide have demonstrated binding in the nucleus accumbens^18^. Furthermore, infusion of Exendin-4 in NAc has been shown to attenuate alcohol self-administration and related behaviors in mice and rats^15,31^.

While GLP-1 agonists have been shown to have central nervous system action, another possible mechanism is slowed gastric emptying^32–34^, which could increase fullness and reduce alcohol consumption. This could explain the prevalence of “nausea” and “appetite suppression” terms in our social media study (Cluster 3; Suppl. Figs. 3 and 4). Additionally, oxidation of alcohol to acetaldehyde can occur in the stomach^35^, potentially explaining the increase in negative effects of alcohol reported in the social media study. In contrast, intraduodenal ethanol rapidly increases blood alcohol levels^36^. Therefore, slowed gastric emptying from the stomach to the upper intestine (duodenum) observed after GLP-1 agonist administration could lead to increased levels of acetaldehyde and an altered or blunted rise in blood alcohol content (BAC). Altering the rising curve of a drug greatly decreases its subjective effects and abuse potential^37^. A decrease in the rewarding effects of alcohol could be driving the decrease in AUDIT scores and drinks per week observed in our remote study. Reduced BAC could also explain the decreased sedative and stimulating effects reported by the participants, here. However, some reports suggest that gastric emptying may return to baseline levels over time^38^. Further mechanistic studies are needed to test these hypotheses and explore potential mechanisms of action of GIP analogues, such as Tirzepatide tested here, for reducing alcohol intake.

### Limitations

The self-report nature of both our studies is subject to selection bias. People experiencing particular effects on alcohol intake, whether positive or negative, are more likely to post about them than those experiencing none. Similarly, participants may have found a decrease in alcohol intake very salient and remembered drinking more before starting their medication. However, given our effects with two classes of drugs as well as cross sectional, between subjects findings, the decrease in drinking observed here is unlikely due to reporting biases alone. Our sample was largely white and female, further studies in more diverse populations are needed to examine sex and race differences. Here, we recruited only participants with obesity in the remote study, limiting our results to this specific population. Future research should investigate the effects of GLP-1 and GLP-1/GIP agonists on alcohol consumption in different weight ranges and between different drug classes. We also report similar levels of alcohol reduction across medication doses (Suppl. Fig. 6), future studies examining optimal dose for alcohol reduction are needed.

## Conclusions

Here we provide a principled analysis of social media posts detailing a reduction in alcohol consumption while taking GLP-1 and Tirzepatide medication. In addition, we report a reduction in average number of drinks, binge drinking, AUDIT score, and the sedative/stimulating effects of alcohol in individuals taking Semaglutide or Tirzepatide in our remote study. These findings add to a growing literature detailing a reduction in alcohol intake after GLP-1 agonist medications. To our knowledge this is the first report of decreased alcohol consumption following Tirzepatide use. Further RCTs are needed to fully explore the therapeutic potential of GLP-1 agonists and GIP/GLP-1 combination drugs for the treatment of Alcohol Use Disorder.

## Methods

### Study 1: social media analysis

#### Data collection

**Figure 7:**
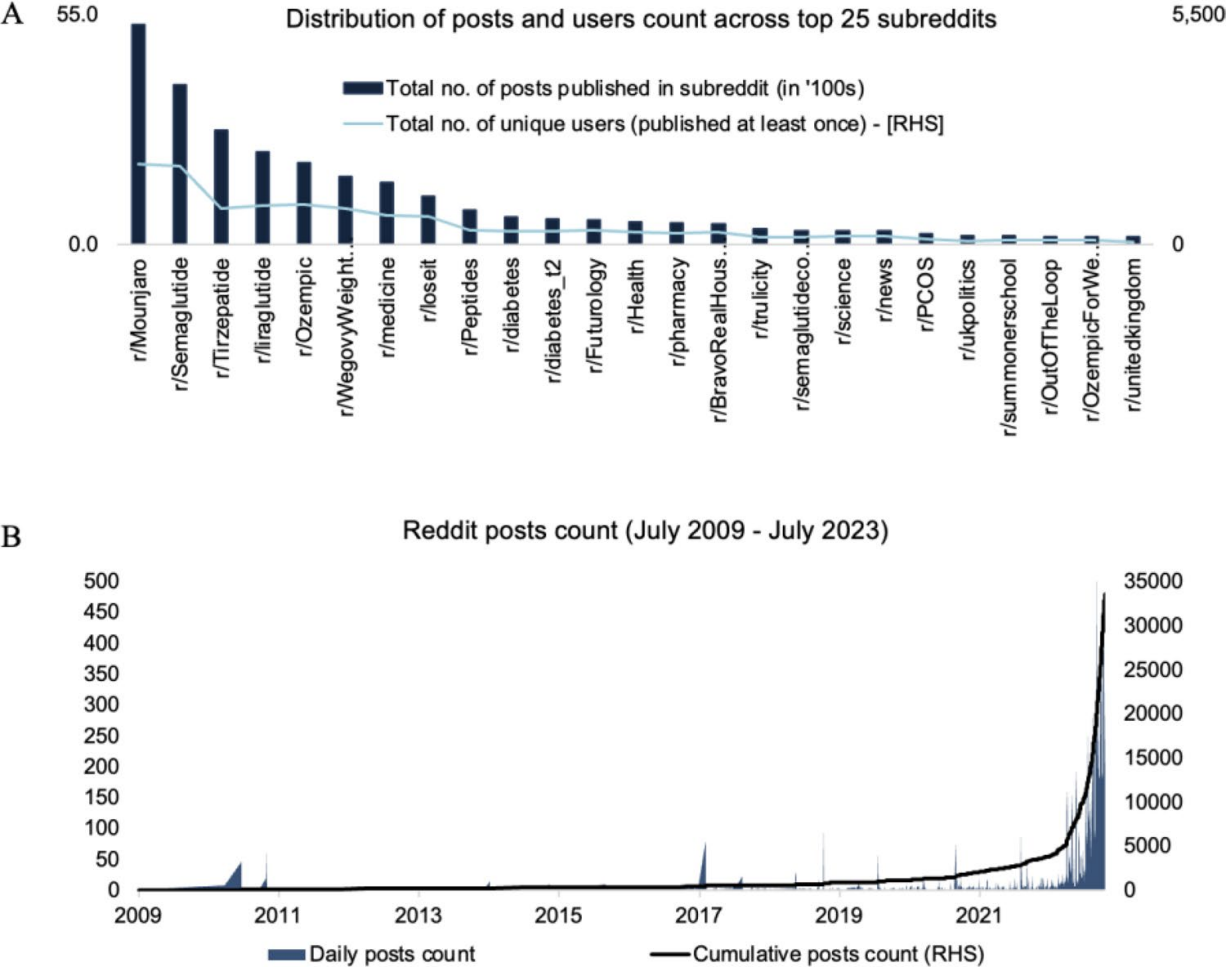
Data exploration and finalization. (**A**) Distribution of posts and users across top 25 subreddits. (**B**) Time-series of daily post count and cumulative post count between 2009 and 2023. Overall, we observe a large influx of posts in recent years, with most posts coming from January 2022 to July 2023. These posts largely came from the top 25 subreddits (89% of total posts), notably from subreddits with a focus specifically on GLP-1 and Tirzepatide.

Reddit posts and their comments were extracted using a list of keywords containing all GLP-1 approved medications (i.e., GLP-1, GIP, GLP1, dulaglutide, exenatide, byetta, semaglutide, liraglutide, lixisenatide, rybelsus, mounjaro, tirzepatide, wegovy, ozempic, trulicity) using the Reddit extractor tool available on Apify (apify.com). Reddit extractor tool allows to extract posts and comments using a set of keywords and export the data in multiple formats. 68,250 posts and comments from 2009 to 2023 were extracted from 313 subreddits. The top 25 subreddits contained 89% of the total extracted posts and comments (Fig. 7A), and discussions started to spike closer to 2021 (Fig. 7B).

#### Data preprocessing

The data preprocessing stage is necessary to ensure the dataset’s uniformity and quality for further analysis. Our focus is to identify underlying themes; hence we performed data cleaning by removing punctuation, capitalization, urls, and special characters. Further, for uniformity, the title of the post and body was merged, as the main idea is often written in the title. Subsequently, the text is cleaned further by removing English stop words, punctuation, capitalization, special characters, and duplicate posts. Finally, we removed comments shorter than 100 characters to reduce noise and enhance content depth. Following these steps, the processed file contained 33,609 relevant posts from 14,595 unique users, which was used in subsequent steps.

#### Feature selection

Preprocessed data was then encoded by the Universal Sentence Encoder (USE) 4, a pre-trained state-of-the-art encoder, by TensorFlow^39^. The encoder translates each post and comment into 512 high-dimension, feature vectors to be used later in the analysis. To establish identifiers, each encoding is given an ID from their respective Reddit post/comment.

#### Cluster classification using machine learning

To determine the optimum number of themes discussed, we employed K-means^40^ as an unsupervised machine learning^41^ algorithm using scikit-learn (https://scikit-learn.org/stable/) machine learning library. Feature vectors generated from the previous step were used as an input to train the model and cluster labels are generated as an output. Elbow plot, which identifies the optimum number of clusters based on distortion scores^42^, was visualized using scikit-yb library (https://www.scikit-yb.org/en/latest/) (Suppl. Fig. 2). 13 optimum clusters were reported from the K-means trained model. Further, we inspected the top 100 unigrams and top 50 bigrams within each cluster. Based on our inspection, we merged clusters based on thematic similarities, finalizing an optimum 8 clusters which encapsulated the majority of discussions.

As a second step, we trained a supervised machine learning model to determine important features and their weights in each cluster. In each iteration of training the Random Forest^43^ based binary classifier, each cluster was separately set to 1, while the rest were labeled 0 to identify the most important features driving the discussions underlying that cluster.

#### Data exploration and visualization

In order to visualize our clusters, we use Uniform Manifold Approximation and Projection (UMAP)^44^ which turns our 512 dimensioned embeddings data into a visualizable, two-dimensional representation. Organization within the UMAP graphs indicates that there is an underlying structure within the clusters. Next, we generate word clouds for the top 30 unigrams and bigrams to reveal visually underlying structure or relationships within the clusters. Finally, we ran a hypergeometric test^45^ for enrichment analysis of alcohol-related posts in all clusters. Next, we inspected all alcohol related posts within clusters identified by the enrichment analysis, using word trees to evaluate the attitude and feelings of individuals on GLP-1 medications towards alcohol. As an additional validation step, we manually inspected 10% of the alcohol-related posts (10 out of every 100) to understand if these posts were mere speculations. Randomly selected posts made by 962 unique individuals pointed towards first-hand experiences related to alcohol as also evident by the word trees (Figs. 3 and 4).

#### Full order partial correlation analysis

A full order partial correlation analysis^46^ was carried out to understand the strength of relationship between clusters; as it has the ability to show a direct relationship between two clusters. To this end, we analyzed 28 combinations, iterating over one combination at a time accounting for 8 optimized clusters.

### Study 2: remote study

#### Participants and procedures

Individuals were recruited from social media including Facebook, Instagram and Reddit across the United States using flyers and paid ads and completed a one-session web-based survey administered on Qualtrics. Inclusion criteria included being: (a) a current alcohol drinker; (b) ≥ 21 years old; (c) a BMI ≥ 30; and (d) (i) either reported taking their current dose for ≥ 30 days of Semaglutide/Tirzepatide or (ii) did not report taking these medications (as a control group) for diabetes or weight loss. Participants completed original and adapted forms of validated assessments (see Supplemental document). In the group with medications, adapted forms of validated assessments were used to investigate differences prior to and while on the current dose of their medication. However, the control group (i.e., individuals with obesity not on these medications to treat type-2 diabetes or weight loss) reported these assessments for the current time point only. The study was approved by VT IRB, and informed consent was obtained from all participants prior to their participation in the study. All methods were performed in accordance with the relevant guidelines. Compensation was provided through a lottery system where 1 randomly selected participant per 50 participants received a $50 gift card.

#### Study measures

##### Demographics

Demographic data, such as age, height and weight (to calculate BMI), gender, race, education and ethnicity were assessed by a demographics questionnaire.

##### Alcohol frequency

We assessed alcohol consumption through three distinct questionnaires; Timeline Follow Back (TLFB), Alcohol use disorder identification test (AUDIT), and a subjective open-ended question about drinks per episode of use reading “How many standard drinks (refer to the standard drink picture) do you typically have during one episode of use when drinking alcohol?”. Participants could see a visualization of what a standard drink constituted of, e.g., 12 fl oz of regular beer or 5 fl oz of table wine is equivalent to one standard drink, when completing each measure. (a) Timeline Follow Back (TLFB). Alcohol use was measured through Timeline Follow Back (TLFB). TLFB has undergone rigorous research and examination^47^ and has high test–retest reliability^48^. All participants completed the TLFB for the past 30 days to assess drinking frequency. (b) Alcohol use disorder identification test (AUDIT). AUDIT^49^ consists of a ten-item scale that is designed to measure alcohol consumption, alcohol drinking behavior and alcohol-related problems. Control group participants completed the original AUDIT questionnaire, and the participants on medications (i.e., Semaglutide or Tirzepatide) completed an adapted form asking about each question prior to and while on their medication. (c) a subjective question about alcohol per episode of typical use was also asked to assess alcohol frequency. Similar to AUDIT, only participants taking Semaglutide or Tirzepatide completed questions asking about prior to and while on their medication, while control group participants only answered about their current typical use.

##### The biphasic alcohol effects scale (BAES)

BAES is a 14-item scale that gauges feelings and emotions post alcohol consumption^50^. Participants report stimulative and sedative feelings on a 0–10 scale, 0 being not at all, to 10 being extremely for those select emotions. An example of some of the emotions are; difficulty concentrating, down, elated, energized and excited etc. For the medication group (i.e., those on Semaglutide or Tirzepatide), an adapted version of BAES was utilized to evaluate differences prior to and while on these medications.

### Statistical analysis

All statistical analyses were conducted using R software (version 4.2.2; R Core Team, 2022). An a priori sample size calculation was performed using repeated measures, within factor ANOVA. Using two groups (i.e., Semaglutide and Tirzepatide), two measurements (pre/post), a medium effect size (Cohen’s f = 0.25), alpha of 0.01, and 95% power, we required 76 participants. We extrapolated an equal proportion of control participants to bring the total number of participants required to complete the study to 114. We over-recruited due to unknown levels of data quality. Participant characteristics were described using mean (standard deviation) and frequencies, and compared between groups using a t-test and Chi-square test where appropriate. For each of our outcomes of interest, a repeated measure within-between interaction was performed with a group, time (i.e., pre/post), and group by time interaction as the explanatory variables and a random effect for the individual was performed using the lme4 package ^51^. The outcome measures of interest include average drinks (Poisson distribution) and binge drinking (Binomial distribution) calculated from TLFB, AUDIT scores (Gaussian distribution), alcohol use per episode of use (Gaussian distribution) and the sedative and stimulative scores from the BAES scale (Gaussian distribution). For each outcome variable, the optimal model was considered to be the model with the lowest Bayesian Information Criterion (BIC). Results from our models are reported as odds ratios (Binomial), incidence rate ratios (Poisson) with 95% confidence intervals, and parameter estimates (Gaussian) with standard error of the means. Significance level was defined as p < 0.05 for all analysis.

### Supplementary Information

Supplementary Information 1.

Supplementary Information 2.

## Data Availability

The raw data and code is available from the corresponding author (wkbickel@vtc.vt.edu) on reasonable request.

## Code availability

The raw data cannot be shared publicly as per Reddit policy, and is available from the corresponding author (wkbickel@vtc.vt.edu) on reasonable request. The code used to produce the results is made available in a public repository. https://github.com/fatimaquddos/GLP1.
