# Supplementary material for: Semaglutide and Tirzepatide reduce alcohol consumption in individuals with obesity
Source: Sci Rep. 2023 Nov 28;13:20998. doi: 10.1038/s41598-023-48267-2 (PMC10684505; doi:10.1038/s41598-023-48267-2)
Supplement: Supplementary file 1 — Supplementary Information 1. [file 41598_2023_48267_MOESM1_ESM.pdf]

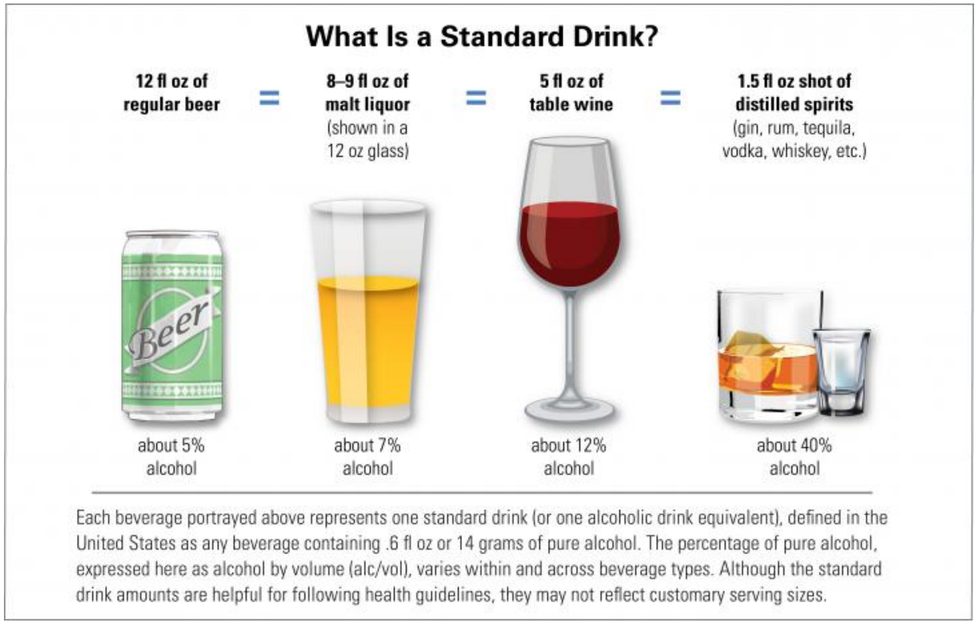

Before starting your **current** dose of **${e://Field/one_medication_other}${e://Field/one_medication_sema}**, how many standard drinks (see the picture above) did you typically have during one episode o drinking alcohol?

After starting your **current** dose of **${e://Field/one_medication_other}${e://Field/one_medication_sema}**, how many standard drinks (see the picture above) do you typically have during one episode of us alcohol?

AUDIT_prepost

How often do you have a drink containing alcohol?

	BEFORE you started your **current** dose of ${e://Field/one_medication_other}${e://Field/one_medication_sema}	AFTER you started your **current** dose of ${e://Field/one_medication_other}${e://Field/one_med
Never	○	○

	BEFORE you started your **current** dose of ${e://Field/one_medication_other}${e://Field/one_medication_sema}	AFTER you started your **current** dose of ${e://Field/one_medication_other}${e://Field/one_med
Monthly or less	○	○
Two to four times a month	○	○
Two to three times per week	○	○
Four or more times a week	○	○

How many drinks did you have on a typical day when you were drinking?

	BEFORE you started your **current** dose of ${e://Field/one_medication_other}${e://Field/one_medication_sema}	AFTER you started your **current** dose of ${e://Field/one_medication_other}${e://Field/one_med
1 or 2	○	○
3 or 4	○	○
5 or 6	○	○
7 to 9	○	○
10 or more	○	○

How often do you have six or more drinks on one occasion?

	BEFORE you started your **current** dose of ${e://Field/one_medication_other}${e://Field/one_medication_sema}	AFTER you started your **current** dose of ${e://Field/one_medication_other}${e://Field/one_med
Never	○	○
Less than monthly	○	○
Monthly	○	○
Weekly	○	○
Daily or almost daily	○	○

How often during the last year have you found that you were not able to stop drinking once you had started?

	BEFORE you started your **current** dose of ${e://Field/one_medication_other}${e://Field/one_medication_sema}	AFTER you started your **current** dose o ${e://Field/one_medication_other}${e://Field/one_me
Never	○	○
Less than monthly	○	○
Monthly	○	○
Weekly	○	○
Daily or almost daily	○	○

How often during the last year have you failed to do what was normally expected of you because of drinking?

	BEFORE you started your **current** dose of ${e://Field/one_medication_other}${e://Field/one_medication_sema}	AFTER you started your **current** dose o ${e://Field/one_medication_other}${e://Field/one_me
Never	○	○
Less than monthly	○	○
Monthly	○	○
Weekly	○	○
Daily or almost daily	○	○

How often during the last year have you needed a first drink in the morning to get yourself going after a heavy drinking session?

	BEFORE you started your **current** dose of ${e://Field/one_medication_other}${e://Field/one_medication_sema}	AFTER you started your **current** dose o ${e://Field/one_medication_other}${e://Field/one_me
Never	○	○
Less than monthly	○	○
Monthly	○	○
Weekly	○	○
Daily or almost daily	○	○

How often during the last year have you had a feeling of guilt or remorse after drinking?

	BEFORE you started your **current** dose of ${e://Field/one_medication_other}${e://Field/one_medication_sema}	AFTER you started your **current** dose o ${e://Field/one_medication_other}${e://Field/one_me
Never	○	○
Less than monthly	○	○
Monthly	○	○
Weekly	○	○
Daily or almost daily	○	○

How often during the last year have you been unable to remember what happened the night before because of your drinking?

	BEFORE you started your **current** dose of ${e://Field/one_medication_other}${e://Field/one_medication_sema}	AFTER you started your **current** dose o ${e://Field/one_medication_other}${e://Field/one_me
Never	○	○
Less than monthly	○	○
Monthly	○	○
Weekly	○	○
Daily or almost daily	○	○

Have you or someone else been injured because of your drinking?

	BEFORE you started your **current** dose of ${e://Field/one_medication_other}${e://Field/one_medication_sema}	AFTER you started your **current** dose o ${e://Field/one_medication_other}${e://Field/one_me
No	○	○
Yes, but not in the last year	○	○
Yes, during the last year	○	○

Has a relative, friend, doctor, or other health care worker been concerned about your drinking or suggested you cut down?

	BEFORE you started your **current** dose of ${e://Field/one_medication_other}${e://Field/one_medication_sema}	AFTER you started your **current** dose o ${e://Field/one_medication_other}${e://Field/one_me
No	○	○
Yes, but not in the last year	○	○
Yes, during the last year	○	○

BAES before

The following adjectives describe feelings that some people have after drinking alcohol. Please rate the extent to which drinking alcohol has produced these feelings in you started taking your current dose of **${e://Field/one_medication_other}${e://Field/one_medication_sema}**.

	**Not at all (0)**	1	2	3	4	5	6	7	8	9
Difficulty Concentrating	○	○	○	○	○	○	○	○	○	○
Down	○	○	○	○	○	○	○	○	○	○
Elated	○	○	○	○	○	○	○	○	○	○
Energized	○	○	○	○	○	○	○	○	○	○
Excited	○	○	○	○	○	○	○	○	○	○
Heavy head	○	○	○	○	○	○	○	○	○	○
Inactive	○	○	○	○	○	○	○	○	○	○
Sedated	○	○	○	○	○	○	○	○	○	○
Slow thoughts	○	○	○	○	○	○	○	○	○	○
Sluggish	○	○	○	○	○	○	○	○	○	○
Stimulated	○	○	○	○	○	○	○	○	○	○
Talkative	○	○	○	○	○	○	○	○	○	○

	Not at all (0)	1	2	3	4	5	6	7	8	9
Up	○	○	○	○	○	○	○	○	○	○
Vigorous	○	○	○	○	○	○	○	○	○	○
	Not at all (0)	1	2	3	4	5	6	7	8	9

BAES after

The following adjectives describe feelings that some people have after drinking alcohol. Please rate the extent to which drinking alcohol has produced these feelings in you started taking your current dose of **${e://Field/one_medication_other}${e://Field/one_medication_sema}**.

	Not at all (0)	1	2	3	4	5	6	7	8	9
Difficulty Concentrating	○	○	○	○	○	○	○	○	○	○
Down	○	○	○	○	○	○	○	○	○	○
Elated	○	○	○	○	○	○	○	○	○	○
Energized	○	○	○	○	○	○	○	○	○	○
Excited	○	○	○	○	○	○	○	○	○	○
Heavy head	○	○	○	○	○	○	○	○	○	○
Inactive	○	○	○	○	○	○	○	○	○	○
Sedated	○	○	○	○	○	○	○	○	○	○
Slow thoughts	○	○	○	○	○	○	○	○	○	○
Sluggish	○	○	○	○	○	○	○	○	○	○
Stimulated	○	○	○	○	○	○	○	○	○	○
Talkative	○	○	○	○	○	○	○	○	○	○
Up	○	○	○	○	○	○	○	○	○	○
