# Supplementary material for: Semaglutide and Tirzepatide reduce alcohol consumption in individuals with obesity
Source: Sci Rep. 2023 Nov 28;13:20998. doi: 10.1038/s41598-023-48267-2 (PMC10684505; doi:10.1038/s41598-023-48267-2)
Supplement: Supplementary file 2 — Supplementary Information 2. [file 41598_2023_48267_MOESM2_ESM.pdf]

Semaglutide and Tirzepatide Reduce Alcohol Consumption in Individuals with Obesity

Fatima Quddos[2,1], Zachary Hubshman[3,1], Allison Tegge[1], Daniel Sane[3,1], Erin Marti[1], Anita S. Kablinger[4], Kirstin M. Gatchalian[1], Amber L. Kelly[2,1], Alexandra G. DiFeliceantonio[1], Warren K. Bickel*[1]

[1] Fralin Biomedical Research Institute at VTC, Virginia Tech, Roanoke, VA, United States

[2] Graduate Program in Translational Biology, Medicine, and Health, Blacksburg, VA, USA

[3] Virginia Polytechnic Institute and State University, Blacksburg, VA, USA

[4] Virginia Tech Carilion School of Medicine, Roanoke, VA, USA.

Supplementary data:

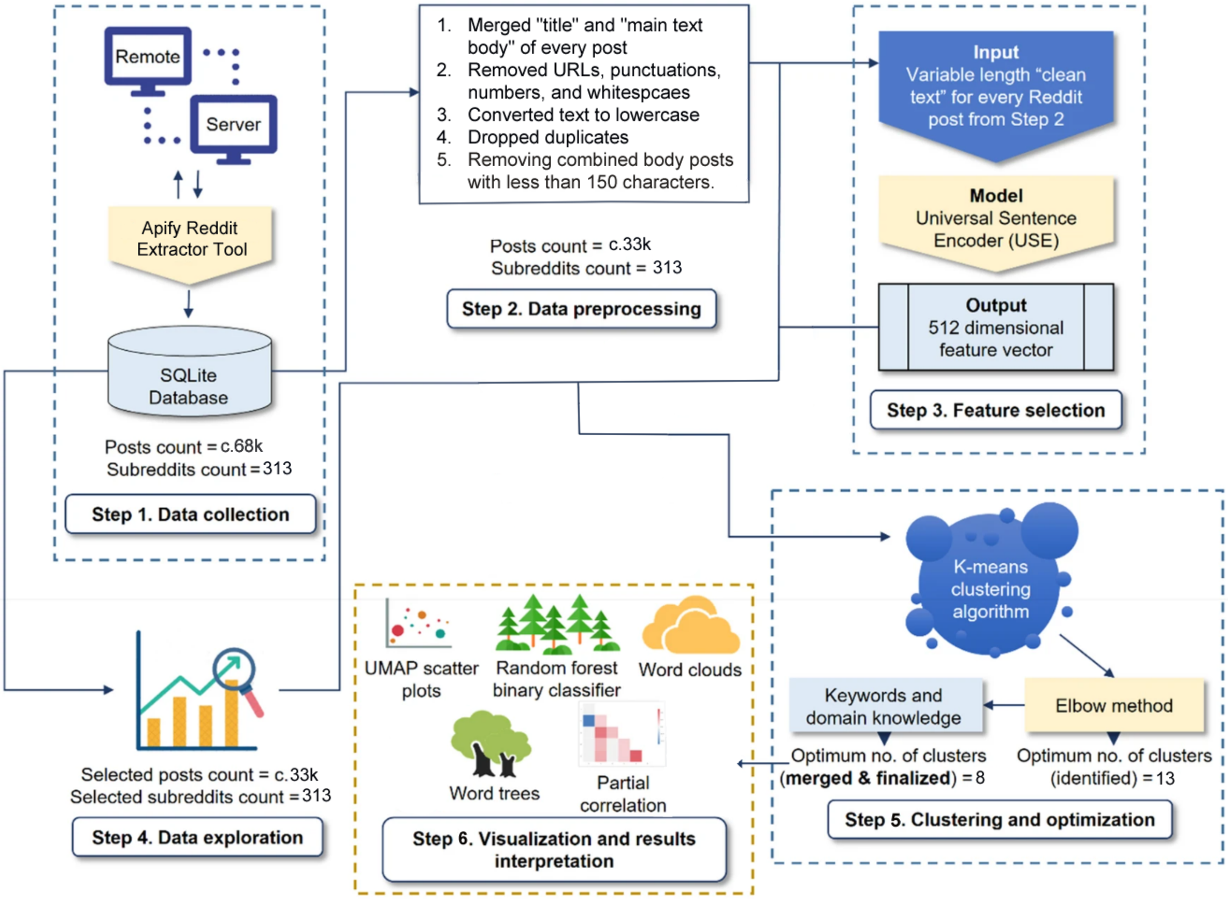

Supplementary Figure 1: A step by step workflow of the social media analysis. The workflow delineates each step and its sequence; starting from Data collection to Visualization and results interpretation. Direction of the workflow is depicted by solid lines with arrows and the dotted squares represent the boundaries of each step.

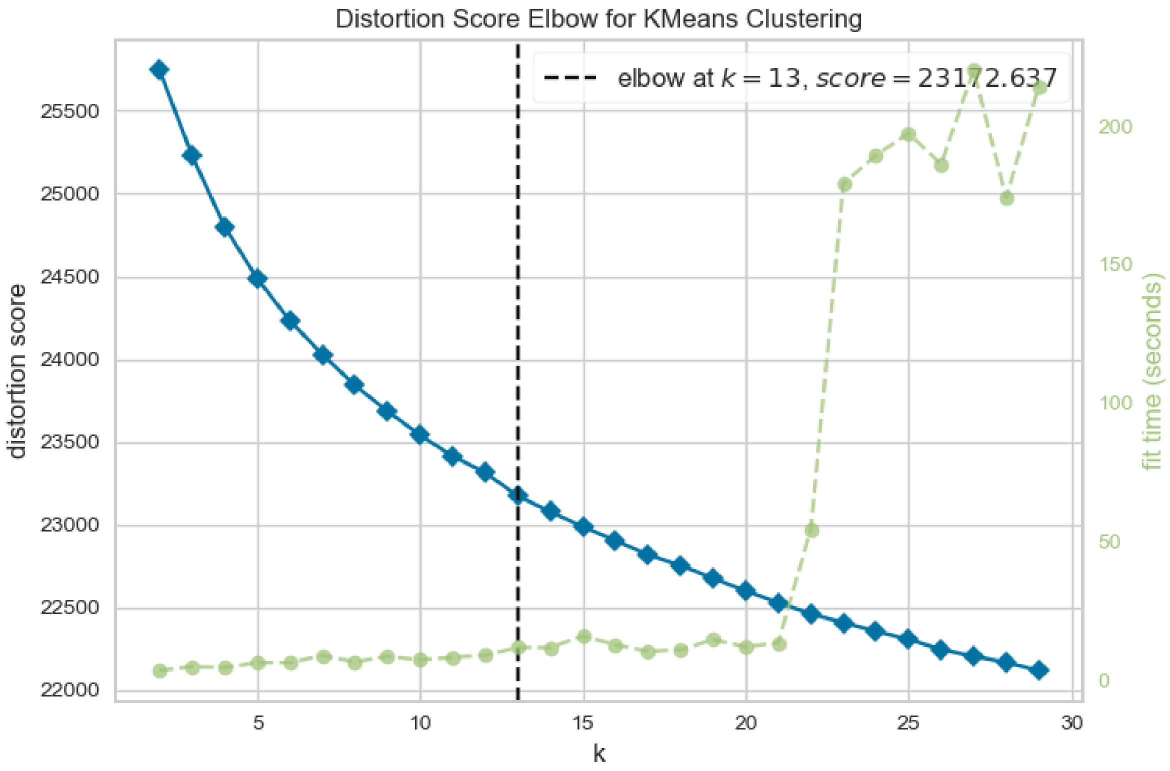

Supplementary Figure 2: Elbow plot. Elbow plot, plotting the distortion scores as a function of the number of clusters, using the elbow method. The heuristic technique determined 13 optimum clusters.

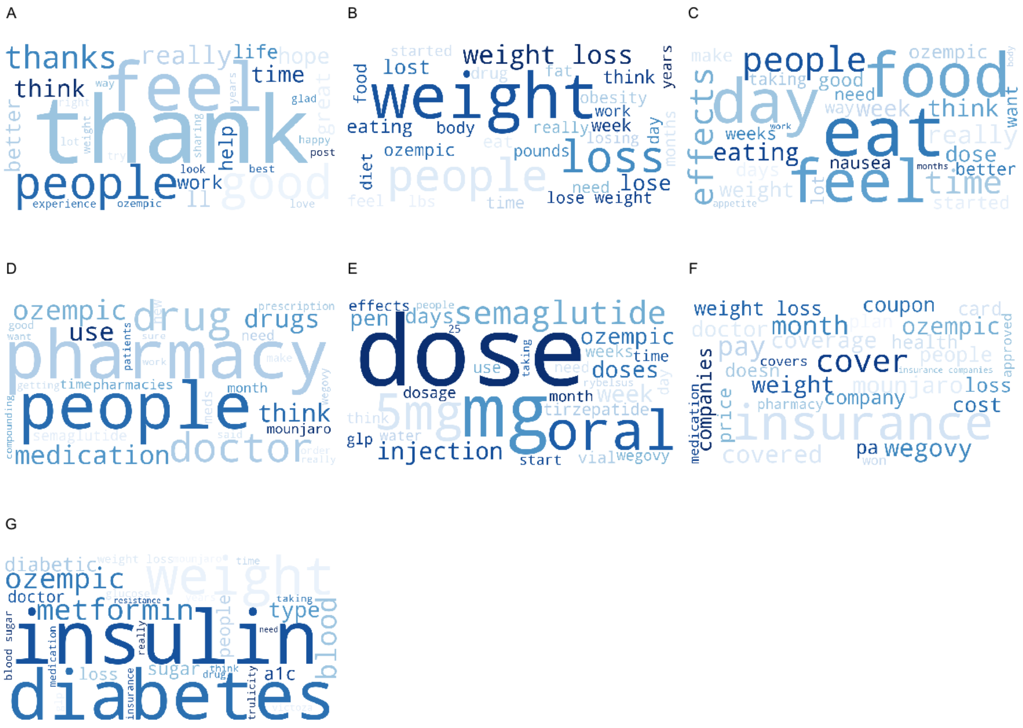

Supplementary Figure 3: Final optimized cluster in word clouds. Word clouds from a given optimized cluster have been generated from the top 30 unigrams, as sorted by Tfidf-Vectorizer weights. This provides insight into the underlying keywords within the 7 main clusters (A-G). We observe that our themes broadly capture the main discussions, as evident by the keywords.

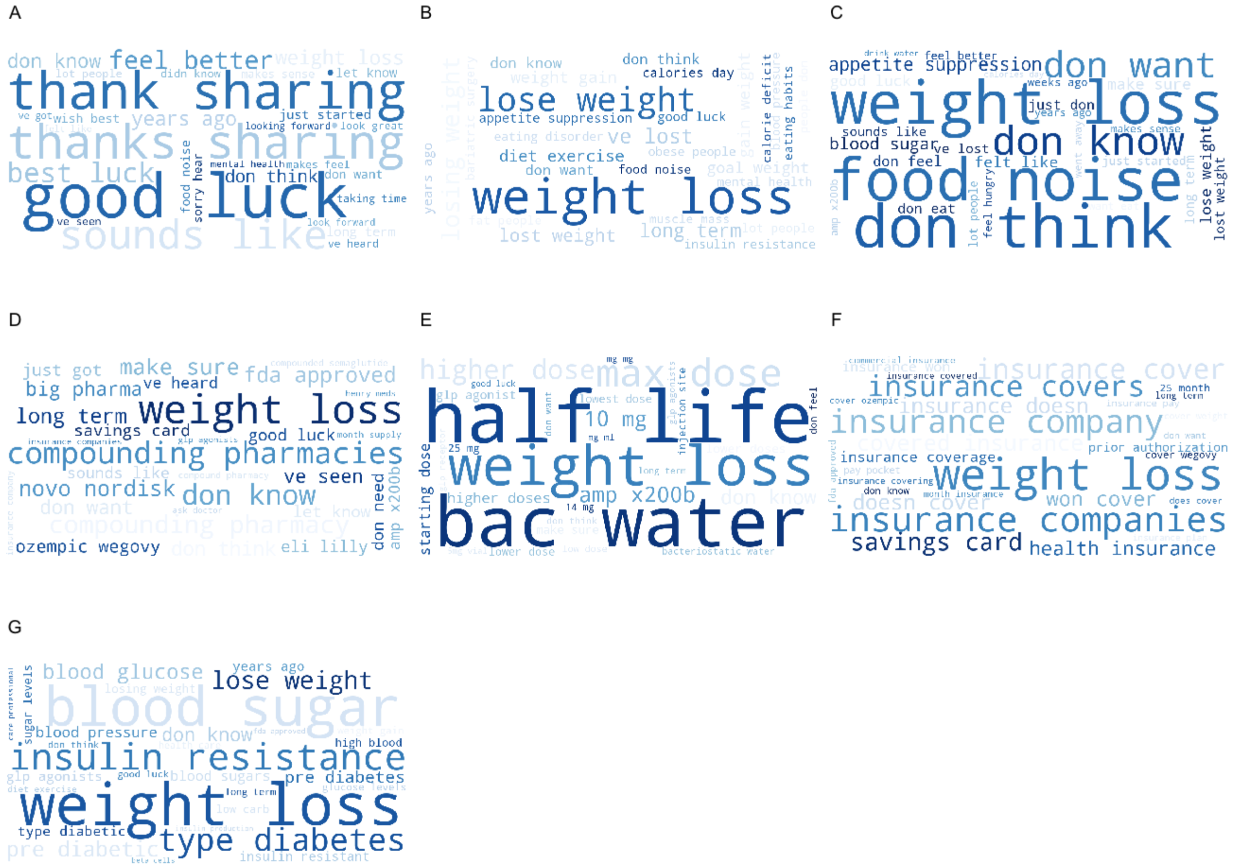

Supplementary Figure 4: Final optimized cluster in word clouds. Word clouds from a given optimized cluster have been generated from the top 30 bigrams, sorted by weights provided by Tfidf-Vectorizer. This provides insight into the underlying keywords within the 7 clusters(A-G). We see distinguishable characteristics within each cluster, interestingly the bigram "weight loss" appears within nearly every cluster (B-G), however on further inspection of other bigrams, we see other words dominate their respective cluster. Due to the informal nature of social media platforms, some bigrams may come off as confusing and unrelated to its cluster. To this end, we will translate notable bigrams from each cluster for a better understanding of underlying key terms.
Cluster A: don - don't
Cluster B: Food noise - Constant thoughts about food
Cluster D: Novo Nordisk + Eli Lilly - Pharmaceutical Companies
Cluster E: BAC Water - Bacteriostatic water
Cluster F: won - won't, doesn - doesn't

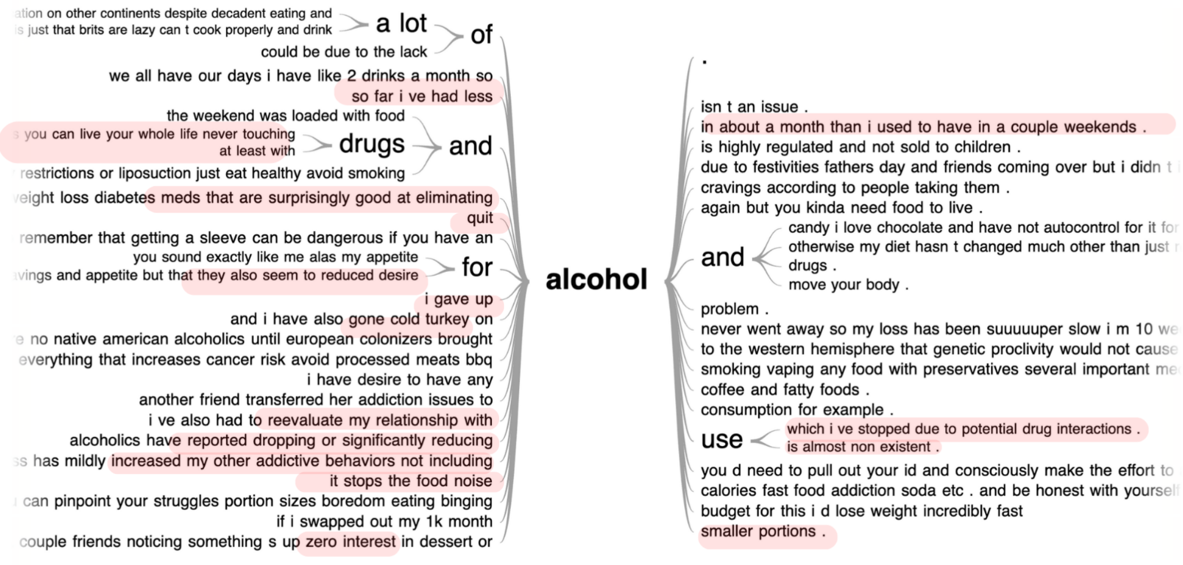

Supplementary Figure 5: Word tree from Cluster 4 generated for alcohol. Any phrases related to a decrease/change in alcohol consumption or effects on alcohol are highlighted with red.

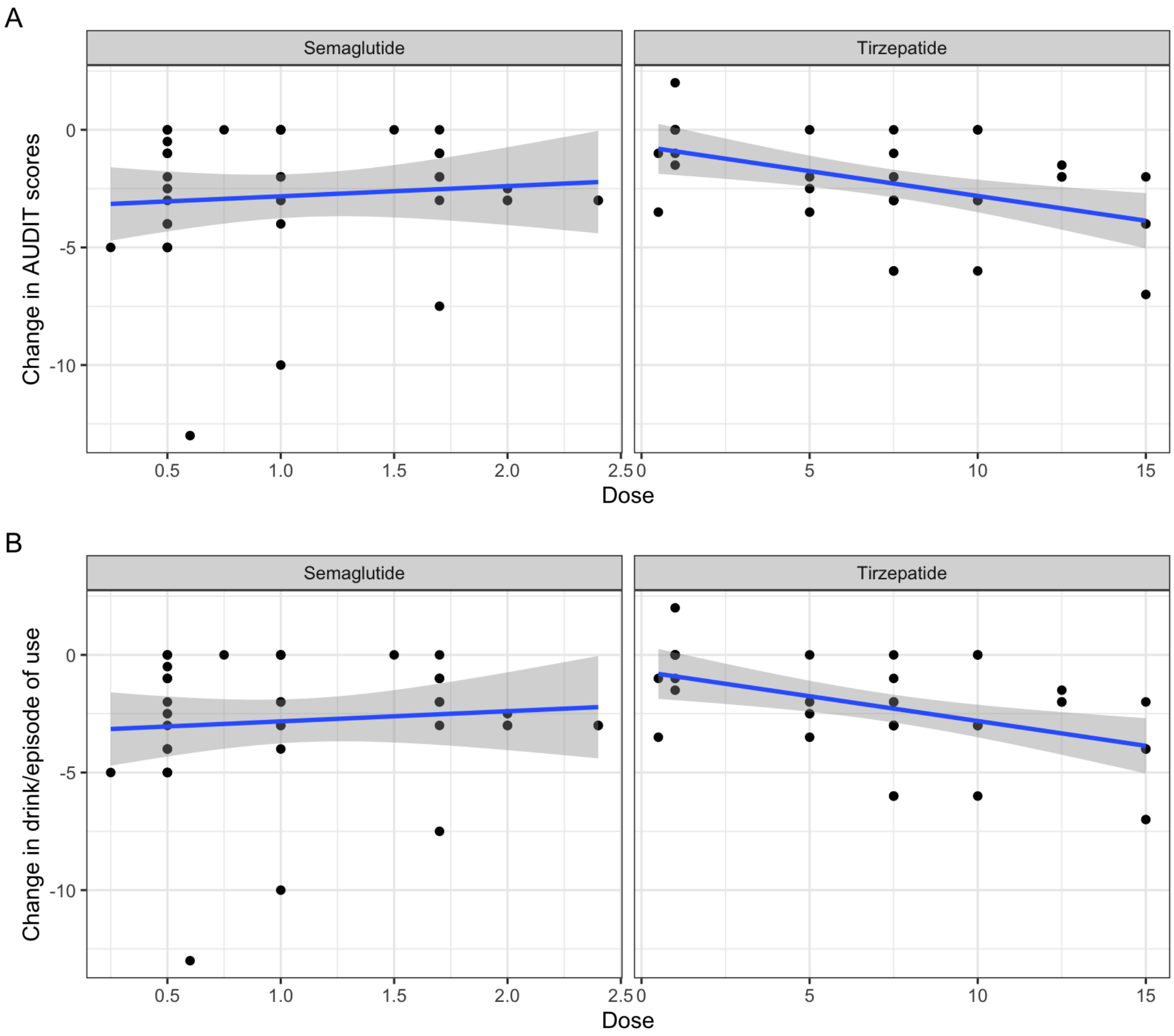

Supplementary Figure 6: - Change in AUDIT scores and drinks per episode of use as a function of dose. In both Semaglutide and Tirzepatide, there is no significant association between dose and change in AUDIT scores or dose and change in drinks per episode of use, although a visual downward trend can be observed in the Tirzepatide group. Change in AUDIT score and drinks per episode per use is calculated by subtracting each value after starting medications from prior to starting medications value.

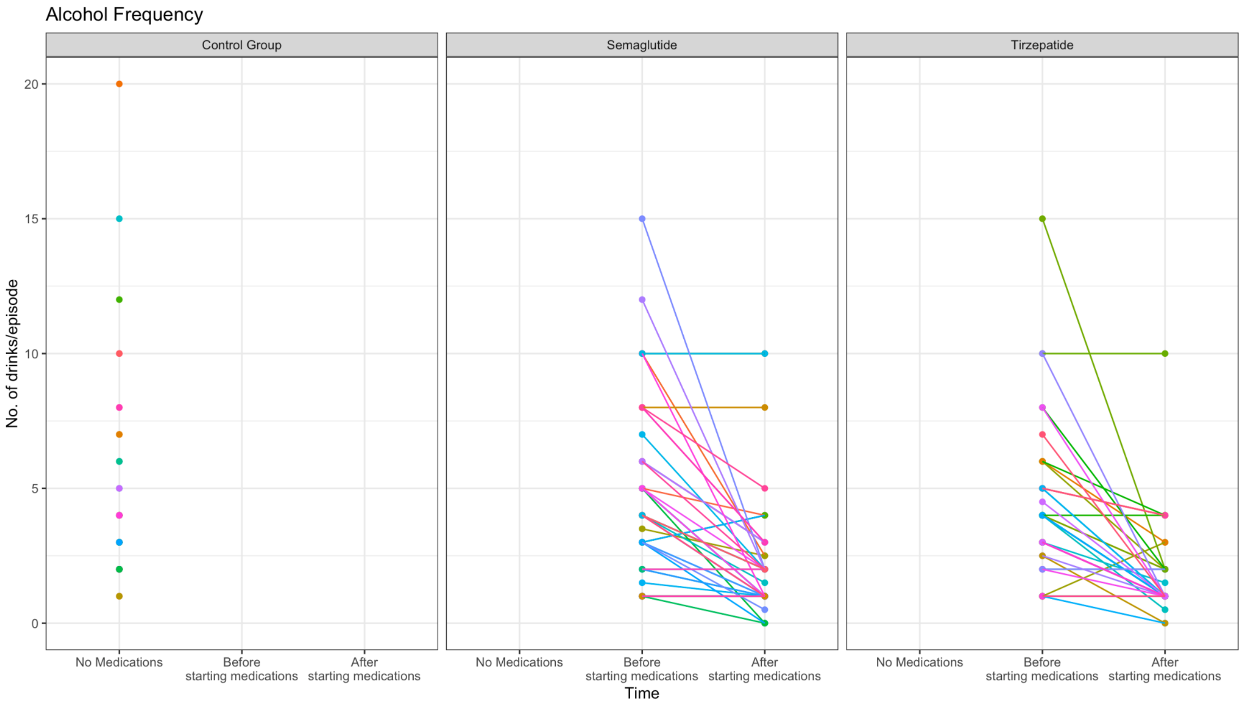

Supplementary figure 7: Individual level data for number of drinks per episode of use faceted by each group.

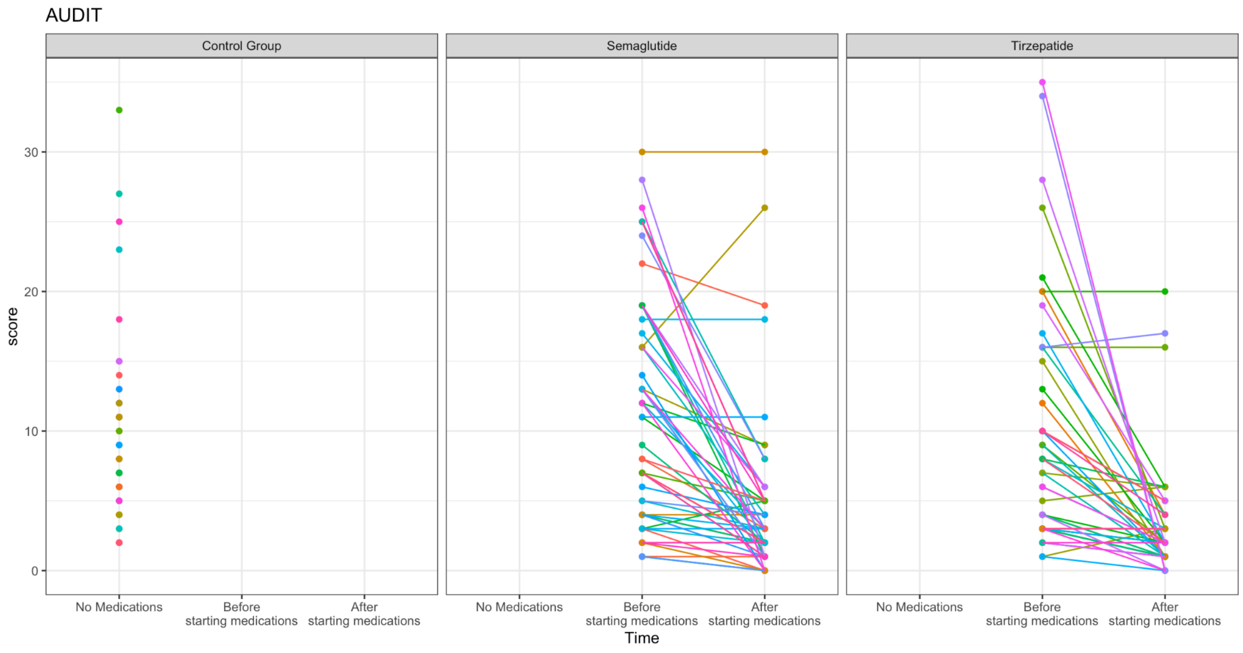

Supplementary figure 8: Individual level data for AUDIT scores faceted by each group.

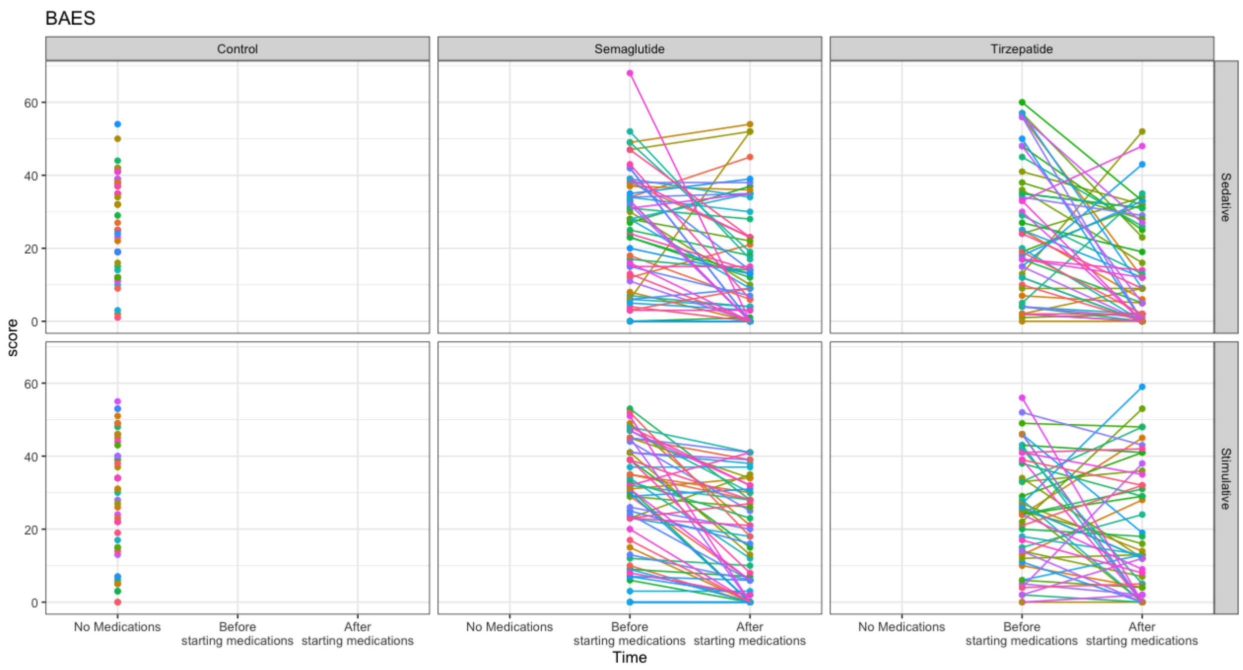

Supplementary figure 9: Individual level data for sedative and stimulative scores of BAES faceted by groups.

Supplementary table 1: Dose (mg) of each medication reported by participants in the remote study.

Medication	Dose (mg)																
	0.25	0.5	0.6	0.75	1	1.5	1.7	2	2.4	2.5	5	7	7.5	10	12.5	14	15
Mounjaro (Tirzepatide)	0	3	0	0	10	0	0	1	0	1	7	0	12	6	3	0	5
Ozempic or Wegovy	2	19	1	1	13	2	9	5	2	0	0	0	0	0	0	0	0
Rybelsus	0	0	0	0	0	0	0	0	0	0	0	1	0	0	0	1	0
